# Nonspecific Effects of Oral Polio Vaccine on Diarrheal Burden and Etiology Among Bangladeshi Infants

**DOI:** 10.1093/cid/cix354

**Published:** 2017-04-24

**Authors:** Alexander Upfill-Brown, Mami Taniuchi, James A. Platts-Mills, Beth Kirkpatrick, Stacey L. Burgess, M. Steven Oberste, William Weldon, Eric Houpt, Rashidul Haque, K. Zaman, William A. Petri

**Affiliations:** 1 Center for World Health, David Geffen School of Medicine at University of California, Los Angeles (UCLA);; 2 Division of Infectious Diseases and International Health, Department of Medicine, University of Virginia, Charlottesville;; 3 Vaccine Testing Center and Unit of Infectious Diseases, Department of Medicine, University of Vermont College of Medicine, Burlington;; 4 Centers for Disease Control and Prevention, Atlanta, Georgia;; 5 Center for Vaccine Science and Parasitology Lab, International Centre for Diarrhoeal Disease Research, Bangladesh, Dhaka

**Keywords:** OPV, vaccines, enteropathogens, diarrhea, cross-protection

## Abstract

**Background.:**

As the global polio eradication initiative prepares to cease use of oral polio vaccine (OPV) in 2020, there is increasing interest in understanding if oral vaccination provides non-specific immunity to other infections so that the consequences of this transition can be effectively planned for and mitigated.

**Methods.:**

Data were collected from infants in an urban slum in Bangladesh (Mirpur, Dhaka) as part of the performance of rotavirus and oral polio vaccines in developing countries (PROVIDE) study. Following vaccination with trivalent oral polio vaccine (tOPV) at 6, 10, and 14 weeks, infants were randomly assigned to receive tOPV (n = 315) or inactivated polio vaccine (IPV) (n = 299) at 39 weeks. Episodes of diarrhea were documented through clinic visits and twice-weekly house visits through 52 weeks. In sum, 14 pathogens associated with diarrhea were analyzed with TaqMan Array Cards.

**Results.:**

Although the proportion of children experiencing diarrhea was not different between the tOPV and IPV groups (*P* = .18), the number of days with diarrhea (*P* = .0037) and the number of separate diarrheal episodes (*P* = .054) trended lower in the OPV arm. Etiological analysis revealed that male tOPV recipients were less likely to have diarrhea of bacterial etiology (*P* = .0099) compared to male IPV recipients but equally likely to experience diarrhea due to viruses (*P* = .57) or protozoa (*P* = .14). Among the 6 bacterial enteric pathogens tested, only *Campylobacter jejuni*/*coli* detection was significantly reduced in the OPV arm (*P* = .0048).

**Conclusions.:**

Our results suggest that OPV may cause nonspecific reductions in mortality, as has been studied elsewhere, by reducing etiology-specific diarrheal burden. This is likely driven by reductions in bacterial diarrhea. Further study of nonspecific OPV effects before global cessation is supported.

**Clinical Trials Registration.:**

NCT01375647.

## Main point

Oral Polio Virus may cause nonspecific reductions in mortality by reducing etiology-specific diarrheal burden, specifically the number of days with diarrhea. This is likely driven by reductions in bacterial—especially *Campylobacter*, *Shigella*—diarrhea. Similar off-target effects were not identified for rotavirus vaccine.

**(See the Editorial Commentary by Aaby and Benn on pages 420–1.)**

Following the cessation of trivalent oral polio vaccine (tOPV) use in April 2016, the Global Polio Eradication Initiative (GPEI) plans to remove all OPV from routine immunization in 2020. OPV containing type 2 virus (OPV2) was removed from global use in April 2016 as an estimated 2200 to 3800 children have been paralyzed due to OPV2 vaccine-associated paralytic polio (VAPP) and circulating vaccine-derived poliovirus (cVDPV2) because wild poliovirus type 2 (WPV2) was eradicated in 1999 [1].

Although OPV possesses a small but direct risk to infants, there is growing awareness that live attenuated oral vaccines such as OPV may cause nonspecific enhancements in immune response [2]. A recent randomized trial found that a birth dose of OPV tended to reduced mortality, although reductions were only statistically significant in boys [3]. Importantly, this effect was primarily seen in the first several months after vaccination [3]. Nonspecific effects—reductions in mortality or disease incidence independent of the effect of the vaccine of the target disease—have also been found for both Bacille Calmette-Guérin (BCG) [4, 5] and measles vaccines [6]. An observational study of children in Denmark found recent OPV recipients had lower rates of hospital admissions for respiratory infections [7]. Before global OPV cessation, the importance of off-target immune enhancement needs to be better understood.

Mechanisms that could explain these nonspecific effects include trained innate immunity and T-cell mediated cross- reactivity [8, 9]. BCG vaccination has been associated with elevation in markers of innate immunity (cytokines interleukin 1β [IL-1β], interleukin 6 [IL-6], tumor necrosis factor α [TNF-α], interferon γ [IFN-γ]) [10, 11]. In these studies, enhanced activation of human monocytes was found to persist at least 3 months and up to 1-year post BCG vaccination, though at lower levels [10, 11]. Animal studies have uncovered epigenetic alterations—including histone trimethylation—in innate immune pathways associated with cross protection to various pathogens [12, 13].

The performance of rotavirus and oral polio vaccines in developing countries (PROVIDE) study was designed to evaluate the performance of oral vaccines (OPV and rotavirus vaccine) in the low-income communities of Mirpur, Dhaka, and Kolkata [14–17]. Detailed data were collected on diarrheal episodes of enrolled children through 1 year of age. We explored PROVIDE data to assess for the possible contribution of oral vaccines to nonspecific reductions in mortality by focusing on reductions in the burden of diarrhea—the second leading cause of mortality in children under 5 in developing countries [18]. We therefore evaluated the nonspecific effects of OPV versus inactivated polio vaccine (IPV) on total and etiology-specific diarrheal incidence during the 3-month window following vaccination.

## DATA AND METHODS

### Study Design

The PROVIDE study was conducted in the Mirpur area of Dhaka, Bangladesh, and ran from May 2011 to November 2014 [15]. A full description of PROVIDE study design is available in reference [16]. All enrolled children received tOPV at 6, 10, and 14 weeks of age. Children were randomly assigned to receive rotavirus vaccine (Rotarix) scheduled at 10 and 17 weeks. At 39 weeks, children were again randomly assigned to receive tOPV or IPV, and at 52 weeks all children received a tOPV booster. Infants were then equally distributed across from groups depending on receipt of rotavirus vaccine or not at 10 and 17 weeks and receipt of IPV or OPV at 39 weeks. All children received other vaccines according to the Bangladesh Expanded Program on Immunizations, including mealses-rubella bivalent vaccine at 40 weeks. Infants enrolled in the study did not participate in national OPV vaccination campaigns. Diarrheal episodes were recorded through clinic visits and twice-weekly household surveillance visits. For analysis, diarrheal episodes were counted beginning 7 days postvaccination—to attempt to exclude infections that were acquired prior to vaccination—through 52 weeks of age. A parallel analysis of rotavirus vaccine (RV) effects was conducted using surveillance from week 18 through 39 (before supplemental OPV or IPV dose). Separate diarrheal episodes were defined as loose, watery stools more than 72 hours apart. Ethics approval for this study was obtained from the University of Virginia, VA, United States, and the International Center for Diarrheal Disease Research, Bangladesh.

### Molecular Diagnostics

Diarrheal stool samples were collected and analyzed for enteric pathogens using custom-developed TaqMan array cards (TAC) [14, 17]. Nucleic acid was extracted from diarrheal stools using the QIAamp Fast DNA Stool mini kit. Two external controls, MS2 bacteriophage and phocine herpes virus, were spiked in the samples during extraction to monitor nucleic acid extraction and amplification efficiency. Enteric pathogens analyzed included 14 pathogens identified through etiological studies to be significantly associated with diarrhea [19]. (Bacteria: enterotoxigenic *Escherichia coli* (ETEC), *Vibrio cholerae*, enteroinvasive *E. coli* (EIEC)/*Shigella*, *Campylobacter jejuni*/*coli*, enteropathogenic *E. coli* (EPEC), and *Salmonella* spp. Virus: Adenovirus 40/41, Astrovirus, norovirus GII, Sapovirus, Rotavirus. Protazoa: *Cryptosporidium hominus/parvum, Entamoeba histolytica*, *Cyclospora cayetanensis.*) A quantification cycle (Cq) of 35 was used as the analytical limit of detection. For these pathogens, quantitative thresholds could be discerned that were highly predictive of diarrhea in a case-control analysis (Table S1). Using these etiological Cq thresholds, we classified episodes of diarrhea as having a bacterial, viral, or protozoan etiology.

### Statistical Analysis

The association between the type of vaccine received at 39 weeks and the number of diarrheal episodes given at least 1 episode, and the number of diarrheal days given at least 1 day with diarrhea were evaluated using zero-truncated Poisson regression. We used generalized estimating equations (GEEs) to fit a logistic regression model to evaluate associations between vaccine type and presence/absence of enteric pathogens to adjust for multiple samples per individual. First, associations were tested between vaccine received and general diarrheal etiologies (bacterial, viral, protozoan). If a general class of pathogen was significantly associated, specific pathogens of that type were individually analyzed. For significant etiological pathogens identified in this analysis, Cox regression was used to assess the relationship of time to occurrence of pathogen-specific diarrhea with the type of vaccine received; cumulative risk curves were drawn using the Kaplan-Meier method. The same method was used to assess impact of rotavirus vaccine on diarrheal burden and etiology between weeks 18 and 39. All *P*-values were considered statistically significant at a level of .05 using 2-sided tests. Bonferroni correction was used to adjust for multiple hypothesis testing. All analyses were performed in R 3.1.2.

## RESULTS

### Study Population

Initially 700 infants were enrolled during the week following birth. In total, 299 children received IPV and 315 received OPV at 39 weeks. A total number of 661 diarrheal episodes were recorded in the study period, of which 468 (71%) had stools samples collected and molecular analysis conducted. These 468 samples were collected from 293 different participants; 82 participants had 2 samples tested, 27 had 3 samples, 7 had 4 samples and 4 had 5+ samples tested.

Summary statistics of infants receiving either IPV or OPV at 39 weeks of age are presented in Table 1. There was a lower proportion of females in the OPV arm, though this difference was not statistically significant.

**Table 1.** Characteristics of Infants Randomized to Receive tOPV or IPV at Week 39 After Birth, Performance of Rotavirus and Oral Polio Vaccines in Developing Countries Study, Mirpur, Dhaka, Bangladesh, May 2011–November 2014

Variable	OPV Arm	IPV Arm	*P*-value^a^
N	315	299	
Female	44.4%	51.1%	.11
Received RV at 10 + 17 wk	47.9%	53.2%	.22
Polio neutralizing Ab at 18 wk	9.86 ± 1.36	9.88 ± 1.36	.83
Breastfed 1 wk prior	96.8%	96.7%	>.99
Height (cm)			
At birth	48.8 ± 1.7	48.6 ± 1.8	.14
At 39 wk	68.3 ± 2.6	68.3 ± 2.4	.91
Weight (kg)			
At birth	2.80 ± 0.35	2.75 ± 0.38	.09
At 39 wk	7.71 ± 1.12	7.68 ± 1.03	.71
SES, water, sanitation			
Income (1000s Tk)	13.3 ± 10.5	12.8 ± 8.7	.51
Mother uneducated	28.3%	28.8%	.96
Open drain by house	40.0%	40.1%	>.99
Drinking water filtered/boiled	57.8%	61.5%	.39
Toilet/septic tank	53.7%	53.5%	>.99
Pre-39 wk diarrhea			
Any diarrhea	87.0%	88.6%	.63
Bacterial etiology	16.4%	13.0%	.17
*C. jejuni/coli* presence	26.9%	27.2%	.97
*Shigella*/EIEC etiology	6.7%	4.2%	.11

Abbreviations: Ab, antibody; IPV, inactivated polio vaccine; RV, rotavirus vaccine; SES, socioeconomic status; tOPV, trivalent oral polio vaccine.

^**a**^No sex-specific differences were identified across any variables above.

### Diarrhea Surveillance

In the 12-week period following vaccination, 57% (170/299) of children in the IPV group and 63% (197/315) of children in the OPV group were recorded as having any diarrhea (*P* = .18). In those who did report diarrhea, children receiving OPV experienced an average of 5.9 days of diarrhea compared to 6.7 days in the IPV group (*P* = .0037). Similarly, children receiving OPV experienced an average of 1.7 distinct diarrheal episodes, compared to 1.9 in the IPV group (*P* = .054). When genders were analyzed separately, male infants alone had significantly reduced days with diarrhea in the OPV arm: 6.6 days compared to 7.7 days in the IPV arm (*P* = .0025) (Table 2).

**Table 2.** Effect of OPV vs IPV at 39 Weeks on Different Diarrheal Outcomes in Subsequent 12 Weeks

Outcome	OPV Group Avg		IPV Group Avg		
	Est	*n*	Est	*n*	*P*-Value
All infants					
Any occurrence of diarrhea	62.5%	315	56.9%	299	.18
Number of days with diarrhea	5.9	197	6.7	170	.0037^a^
Number of diarrheal episodes	1.7	197	1.9	170	.054^a^
Females					
Any occurrence of diarrhea	65.0%	140	53.6%	153	.062
Number of days with diarrhea	5.2	91	5.5	82	.23^a^
Number of diarrheal episodes	1.6	91	1.7	82	.26^a^
Males					
Any occurrence of diarrhea	60.6%	175	60.3%	146	.99
Number of days with diarrhea	6.6	106	7.7	88	.0025^a^
Number of diarrheal episodes	1.8	106	2.1	88	.10^a^

Abbreviations: IPV, inactivated polio vaccine; OPV, oral polio vaccine.

^a^ *P*-value from zero-truncated Poisson model.

### Diarrheal Pathogens

Next, we examined if OPV affected the general class of pathogen found in diarrheal samples. Episodes were categorized as bacterial, viral, or protozoan using the TAC diagnostic test. In sum, 230 of 468 (49%) diarrheal samples had at least 1 pathogen identified at or above the quantitative polymerase chain reaction (PCR) threshold for a significant diarrheal pathogen: 178 had 1 pathogen, 43 had 2, 7 had 3, and 2 had 4 or more pathogens. Only male children receiving OPV at 39 weeks had a smaller proportion of diarrheal episodes due to bacterial etiologies (*P* = .0099) (Table 3).

**Table 3.** Effect of OPV vs IPV at 39 Weeks on Different Pathogen Classes of Diarrheal Etiology

Likely Etiology	OPV Group Avg		IPV Group Avg		*P*-Value^a^	
	Est	*n*	Est	*n*	Standard	Adjusted
All infants						
Viral	25.9%	251	29.5%	217	.41	>.99
Bacterial	21.9%	251	27.2%	217	.19	.57
Protozoan	4.0%	251	6.0%	217	.37	>.99
Females						
Viral	22.0%	100	35.6%	90	.041	.12
Bacterial	30.0%	100	23.3%	90	.30	.90
Protozoan	6.0%	100	4.4%	90	.67	>.99
Males						
Viral	28.5%	151	25.2%	127	.57	>.99
Bacterial	16.6%	151	30.0%	127	.0099^b^	.030
Protozoan	2.6%	151	7.1%	127	.14	.42

Abbreviations: IPV, inactivated polio vaccine; OPV, oral polio vaccine.

^a^ *P*-values from GEE binomial model with robust standard errors

^b^Statistically significant after Bonferroni adjustment for multiple hypothesis testing

**Figure 1.**
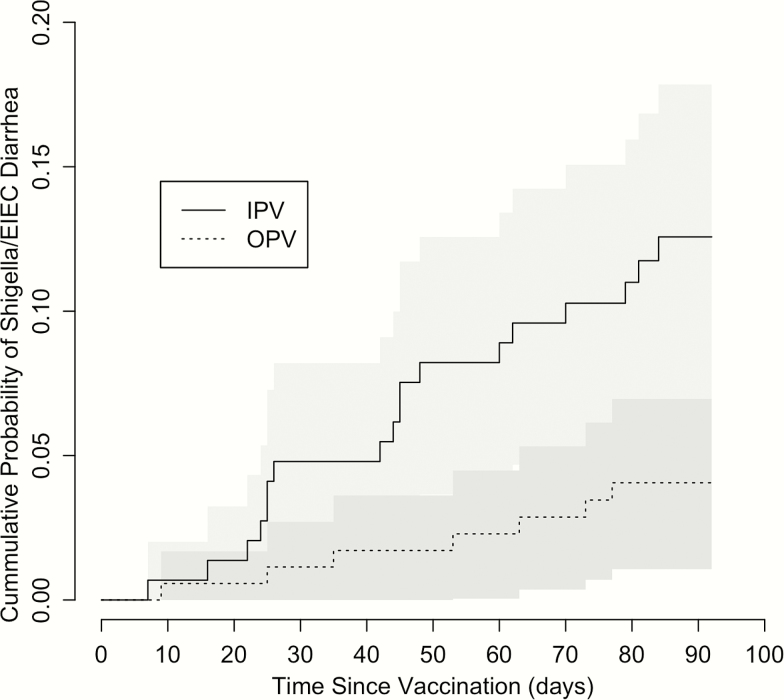
Cumulative probability of experiencing *Shigella*/EIEC diarrhea among male infants receiving OPV or IPV at 39 weeks over subsequent 12-week period. Shaded regions represent 95% confidence interval. Abbreviations: EIEC, enteroinvasive *E. coli*; IPV, inactivated polio vaccine; OPV, oral polio vaccine.

Next, we assessed the individual associations of bacterial pathogens with OPV vaccination at 39 weeks, using the PCR threshold to identify pathogens likely causing diarrhea. *Salmonella* spp., *C. Jejuni/coli* were not included as there were no positives. EPEC was also excluded because of a very small number of positives (4) resulted in model failure. This left 3 bacterial pathogens that were tested. In males, OPV was associated with fewer episodes of *Shigella*/EIEC diarrhea: those receiving IPV were 3.3 times (95% confidence interval [CI]: 1.3–8.6) more likely to have *Shigella*/EIEC diarrhea than children receiving OPV (adjusted *P* = .038). When analyzed from an individual perspective, males receiving OPV had a significantly longer time to first *Shigella*/EIEIC diarrhea (*P* = .009, Figure 1). No other pathogens were significantly related to vaccine received after adjusting for multiple testing (Table S2).

Lastly, we evaluated whether OPV at 39 weeks was associated with differences in the simple presence of bacterial pathogens in stool (i.e., at any detectable concentration). *Salmonella spp*. was not detected at any level in any samples so it was excluded here. OPV vaccination at 39 weeks was associated with a reduction in the detection of any *C. jejuni/coli* in diarrheal stool in all infants: children receiving IPV were 1.9 times (95% CI: 1.2–2.9) more likely to have the bacteria in their diarrheal stool at any quantity (adjusted *P* = .024) (Table S2).

### Rotavirus Vaccine

Infants who received Rotarix vaccine experienced an average of 1.5 fewer days of diarrhea in the 21 weeks following receipt of vaccine compared to those who did not (*P* < .0001, Table S3). The reduction in diarrheal days was greater for males that received RV compared to females that received RV, though reduction was statistically significant for both groups (Table S3). Infants who received RV demonstrated a significant decrease in viral diarrhea (*P* = .004) but not bacterial or protozoan diarrhea (Table S4). When viral pathogens were analyzed separately, RV was associated with a reduction in rotavirus-attributable diarrhea (*P* < .0001) and the presence of rotavirus in diarrhea (*P* = .0001)—however, no other effects were detected (Table S5).

## DISCUSSION

The most important finding of this study is that receiving OPV was associated with significantly fewer days of diarrhea in the 12 weeks after vaccination compared to receiving IPV. Although this difference is small (~ 1 day), we would expect the measured protective effect to be amplified when comparing an IPV-only routine vaccination schedule to an OPV-only one. Furthermore, a longer period of follow-up may also uncover a larger effect of OPV on diarrheal burden.

The work therefore supports the hypothesis that vaccination with the live attenuated Sabin virus may induce off-target immunity toward a subset of diarrheal pathogens [3]. The active diarrheal surveillance in this study allowed such a difference to be detected for the first time. Similar to other studies, we found that male infants experienced greater protective effects that females, possibly due to their higher baseline burden of disease [20]. Although no results regarding female infants were significant after controlling for multiple testing, some parameters suggested females fared worse with OPV (in the case of occurrence of any diarrhea or *shigella* diarrhea specifically) or better with OPV (in the case of diarrhea of viral etiology). A recent study uncovered sex-specific differences in nonspecific immunomodulation following DTP and measles vaccine [21], but further studies of the mechanism underlying these differences is needed.

Nonspecific effects of OPV on diarrhea appear to be limited to diarrhea caused by bacterial pathogens, and in males specifically. When bacterial pathogens were analyzed individually, we found that OPV use was associated with reductions in *C. jejuni/coli* diarrheal prevalence in all infants, and *Shigella*/EIEC diarrheal prevalence at etiological levels when males were analyzed separately. *C. jejuni/coli* and *Shigella* are similar in that they cause inflammatory diarrhea, including dysentery—they are responsible for the vast majority of bacillary dysentery. If OPV given at birth primes an immune response that it is protective against mortality,^3^ and that mortality benefit is seen primarily in the first few months, then it is likely that the protection is against invasive bacterial infection, that is, neonatal sepsis.

Exposure to viral antigens has previously been associated with nonspecific protection from bacterial pathogens. Vaccination with herpes simplex virus type 1 can provide protection from *Listeria monocytogenes* via induction of more robust CD8 T-cell cytotoxicity, prolonged production of IFN-γ, and systemic activation of macrophages in mouse models [22, 23]. OPV can induce long-term cytotoxic CD8 T cell responses and CD4 memory; however, cross-reactivity with bacterial pathogens has not been well explored [24]. As nonimpaired CD4 and T-cell immunity is important for protection of patients from infection with *C. jejuni* perhaps OPV provides for a more robust cross-reactive T-cell response that helps in clearance of *C. jejuni* [25]. Alteration of T-cell responses might be a downstream effect of changes in responsiveness of innate population such as myeloid cells or dendritic cells as observed with BCG vaccination. Increased cytokine production from innate cells in OPV vaccinated patients may help support more robust T-cell responses. OPV vaccination is known to induce type I interferon production from human mononuclear blood cells, and these responses are important for protection from *C. jejuni* [26, 27].

We found no evidence for nonspecific effects of rotarix vaccine on diarrheal burden or etiology. Although RV was associated with a reduction in days with diarrhea, the only pathogen reduced by RV was rotavirus [28]. This may be due to weaker replication of rotavirus vaccine in the gastrointestinal tract and a resulting weakened immune response [29]. The period studied for RV effects was earlier than that for OPV (18 to 39 vs 40 to 52 weeks), so differences in overall immune system development make direct comparison more complicated.

There are several limitations to our study. First, the relatively short period of follow-up (12 weeks) did not allow for analysis of longer term nonspecific effects. Second, the small sample size in this study prevented a formal study of the impact of OPV on all-cause mortality. Third, analysis of diarrheal etiology relied on case-control derived thresholds that are not equally discriminatory for all pathogens, especially *C. jejuni* [19]. This could explain why no association with *C. jejuni* was found at etiological levels. Additionally, we were unable to analyze the effect of OPV on respiratory infections, which has been examined elsewhere and is another leading cause of infant mortality worldwide [7, 18]. Finally, our study compared the impact of OPV versus IPV in infants with a background of 3 OPV doses. This may result in a smaller estimated treatment effects compared to a study in OPV naive children.

We find nonspecific effects of OPV on diarrheal burden, which appear to be limited to diarrhea caused by bacterial pathogens in males. Previous studies of OPV have also examined nonspecific effects; however, these have examined OPV relative to no OPV as opposed to OPV versus IPV [3], as we do here. This comparison is most relevant question for GPEI policy decisions as when OPV is withdrawn from global usage in routine immunization, IPV will be used in its place. Although we find protective effects of OPV relative to IPV here, this study only begins to address the nonspecific trade-offs of OPV compared to IPV. Further research is necessary to understand the differences in OPV-only versus IPV-only routine vaccination schedules in order to fully appreciate the consequences of OPV cessation, currently planned for 2020. Given the risk of reverting to wild-type virus, global usage of OPV must be stopped in order to complete polio eradication. However, better understanding of the unintended negative consequences of OPV withdrawal is necessary so that these can be mitigated.

## Supplementary Data

Supplementary materials are available at *Clinical Infectious Diseases* online. Consisting of data provided by the authors to benefit the reader, the posted materials are not copyedited and are the sole responsibility of the authors, so questions or comments should be addressed to the corresponding author.

## Supplementary Material

Supp_Tables_Revised_18Feb17_clean

## Notes

***Authors’ Contributions.*** A. U. B. led data analysis and writing of the report. M. T. led data acquisition. J. A. P.-M. contributed to data acquisition and data analyses. R. H., B. K., E. H., and W. A. P. designed the original protocol and contributed to data interpretation and writing of the report. M. T., J. A. P.-M., S. L. B., M. S. O., W. W., and K. Z. contributed to interpretation and writing of the report.

***Acknowledgements.*** We thank the children and their families who participated in the PROVIDE study in Mirpur and the Parasitology Lab at icddr,b for sample collection and processing.

***Financial support.*** This work was supported by grants to W.A.P. from the Bill and Melinda Gates Foundation [OPP# 1017093] and National Institutes of Health [R501 A1043596].

***Potential conflict of interests.*** All authors declare no competing interests. All authors have submitted the ICMJE Form for Disclosure of Potential Conflicts of Interest. Conflicts that the editors consider relevant to the content of the manuscript have been disclosed.
