# Supplementary material for: Nonspecific Effects of Oral Polio Vaccine on Diarrheal Burden and Etiology Among Bangladeshi Infants
Source: Clin Infect Dis. 2017 Apr 24;65(3):414–9. doi: 10.1093/cid/cix354 (PMC5848225; doi:10.1093/cid/cix354)
Supplement: Supp_Tables_Revised_18Feb17_clean [file cix354_suppl_supp_tables_revised_18feb17_clean.docx]

**Non-Specific Effects of Oral Polio Vaccine on Diarrheal Burden and Etiology among Bangladeshi Infants: Supplementary Material**

**Table S1** – Quantitative PCR cutoffs used for each pathogen to attribute etiology of diarrheal episode.

| **Pathogen** | **Highly Diarrhea-Associated Quantity (Cq)^1^** |
| --- | --- |
| *Vibrio cholera* | 33·8 |
| Rotavirus | 32·6 |
| *Entamoeba histolytica* | 32.8 |
| *Cyclospora cayetanensis* | 29.6 |
| *Shigella*/EIEC | 27.9 |
| *Salmonella* | 30.7 |
| ST-ETEC (STh) | 22.8 |
| *Helicobacter pylori* | 30.8 |
| Astrovirus | 22.2 |
| *Cryptosporidium* | 24.0 |
| Norovirus GII | 23.4 |
| Adenovirus 40/41 | 22.7 |
| *C. jejuni/C. coli* | 15.4 |
| Typical EPEC (*bfpA*) | 16.0 |
| Sapovirus | NA |
| ^1^ from Liu J, Platts-Mills JA, Juma J, et al. Use of quantitative molecular diagnostic methods to identify causes of diarrhoea in children: a re-analysis of the GEMS case-control study. *Lancet* 2016; **388**:1291–301. | |

**Table S2** – Effect of OPV vs IPV on etiological bacterial pathogens at different Cq thresholds.

| **Cq Threshold** | **Pathogen** | **OPV Group Avg** | **IPV Group Avg** | **P-Value^[[1]](#endnote-1)^** | |
| --- | --- | --- | --- | --- | --- |
| **Etiological** | *All infants (n=468)* |  |  | *Standard* | *Adjusted* |
|  | *Shigella*/EIEC | 10.0% | 12.0% | 0.50 | >0.99 |
|  | *Males (n=278)* |  |  |  |  |
|  | *Shigella*/EIEC | 5.3% | 15.8% | 0.013 | 0.038 |
|  | *Females (n=190)* |  |  |  |  |
|  | *Shigella*/EIEC | 17.0% | 6.7% | 0.028 | 0.056 |
| **Any Detectable** | *All infants* |  |  |  |  |
|  | C. jejuni/coli | 25.1% | 38.7% | 0.0048 | 0.024 |
|  | *Shigella*/EIEC | 19.1% | 22.1% | 0.47 | >0.99 |
|  | *Males* |  |  |  |  |
|  | C. jejuni/coli | 23.8% | 37.0% | 0.038 | 0.19 |
|  | *Shigella*/EIEC | 15.2% | 26.0% | 0.057 | 0.29 |
|  | *Females* |  |  |  |  |
|  | C. jejuni/coli | 27.0% | 41.1% | 0.057 | 0.28 |
|  | *Shigella*/EIEC | 25.0% | 16.7% | 0.14 | 0.69 |
| **Quantitative Relationship (Cq)** | *All infants* |  |  |  |  |
|  | C. jejuni/coli | 27.2 | 26.5 | 0.38 | >0.99 |
|  | *Shigella*/EIEC | 27.9 | 26.8 | 0.32 | >0.99 |
|  | *Males* |  |  |  |  |
|  | C. jejuni/coli | 27.9 | 27.2 | 0.52 | >0.99 |
|  | *Shigella*/EIEC | 29.9 | 26.1 | 0.0044 | 0.02 |
|  | *Females* |  |  |  |  |
|  | C. jejuni/coli | 26.4 | 25.6 | 0.55 | >0.99 |
|  | *Shigella*/EIEC | 26.0 | 28.4 | 0.12 | 0.59 |

1. From GEE model with robust standard errors, with Bonferroni adjustment.

   **Table S3** - Effect of Rotarix vs no vaccine at 10 and 17 weeks on different diarrheal outcomes in subsequent 22 weeks.

   | **Outcome** | | **Rota Group Avg** | | **Control Group Avg** | | **P-Value** |  |  |
   | --- | --- | --- | --- | --- | --- | --- | --- | --- |
   | *All infants* | | *Est* | *n* | *Est* | *n* |  |  |  |
   | Any occurrence of diarrhea | | 80.3% | 310 | 80.9% | 304 | 0.93 |  |  |
   | Number of days w/ diarrhea | | 10.1 | 249 | 11.6 | 246 | <0.0001^1^ |  |  |
   | Number of diarrheal episodes | | 2.4 | 249 | 2.5 | 246 | 0.29^1^ |  |  |
   | *Females* | |  |  |  |  |  |  |  |
   | Any occurrence of diarrhea | | 73.0% | 162 | 78.0% | 159 | 0.40 |  |  |
   | Number of days w/ diarrhea | | 10.0 | 108 | 11.0 | 113 | 0.018^1^ |  |  |
   | Number of diarrheal episodes | | 2.4 | 108 | 2.4 | 113 | 0.96^1^ |  |  |
   | *Males* | |  |  |  |  |  |  |  |
   | Any occurrence of diarrhea | | 87.0% | 162 | 83.6% | 159 | 0.48 |  |  |
   | Number of days w/ diarrhea | | 10.2 | 141 | 12.1 | 133 | <0.0001^1^ |  |  |
   | Number of diarrheal episodes | | 2.4 | 141 | 2.6 | 133 | 0.14^1^ |  |  |
   |  | | ^1^ p-value from zero-truncated Poisson model | | | | | | |

   **Table S4** - Effect of Rotarix vs no vaccine at 10 and 17 weeks on different classes of diarrheal etiology in subsequent 22 weeks

   | **Likely Etiology** | **Rota Group Avg** | | **Control Group Avg** | | **P-Value**^1^ | |
   | --- | --- | --- | --- | --- | --- | --- |
   | *All infants* | *Est* | *n* | *Est* | *n* | *Standard* | *Adjusted* |
   | Viral | 25.2% | 464 | 35.8% | 497 | 0.0004^2^ | 0.0012 |
   | Bacterial | 14.7% | 464 | 15.1% | 497 | 0.85 | >0.99 |
   | Protozoan | 3.7% | 464 | 2.6% | 497 | 0.39 | >0.99 |
   | *Females* |  |  |  |  |  |  |
   | Viral | 21.6% | 190 | 37.9% | 214 | 0.0003^2^ | 0.0009 |
   | Bacterial | 12.1% | 190 | 16.8% | 214 | 0.20 | 0.60 |
   | Protozoan | 4.7% | 190 | 2.3% | 214 | 0.26 | 0.78 |
   | *Males* |  |  |  |  |  |  |
   | Viral | 27.8% | 274 | 34.3% | 283 | 0.10 | 0.30 |
   | Bacterial | 16.4% | 274 | 13.8% | 283 | 0.39 | >0.99 |
   | Protozoan | 2.9% | 274 | 2.8% | 283 | 0.94 | >0.99 |
   | ^1^ p-values from GEE binomial model with robust standard errors  ^2^ Statistically significant after Bonferroni adjustment for multiple hypothesis testing | | | | | | |

   **Table S5** - Effect of Rotarix vaccine versus no vaccine at 10 and 17 weeks on etiological viral pathogens at different Cq thresholds.

   | **Cq Threshold** | **Pathogen** | **Rota Group Avg** | **Control Group Avg** | **P-Value** | |
   | --- | --- | --- | --- | --- | --- |
   | **Etiological** | *All infants (n=442)* |  |  | *Standard* | *Adjusted* |
   |  | *Rotavirus* | 12.9% | 23.5% | 0.00002 | 0.00009 |
   |  | *Males (n=246)* |  |  |  |  |
   |  | *Rotavirus* | 13.7% | 23.3% | 0.004 | 0.017 |
   |  | *Females (n=196)* |  |  |  |  |
   |  | *Rotavirus* | 11.6% | 23.8% | 0.002 | 0.006 |
   | **Any Detectable** | *All infants* |  |  |  |  |
   |  | *Rotavirus* | 19.2% | 27.6% | 0.002 | 0.008 |
   |  | *Males* |  |  |  |  |
   |  | *Rotavirus* | 20.8% | 26.5% | 0.10 | 0.51 |
   |  | *Females* |  |  |  |  |
   |  | *Rotavirus* | 16.8% | 29.0% | 0.003 | 0.016 |
   | **Quantitative Relationship (Cq)** | *All infants* |  |  |  |  |
   |  | *Rotavirus* | 27.4 | 24.0 | 0.00004 | 0.0002 |
   |  | *Males* |  |  |  |  |
   |  | *Rotavirus* | 26.7 | 23.9 | 0.011 | 0.056 |
   |  | *Females* |  |  |  |  |
   |  | *Rotavirus* | 28.7 | 24.1 | 0.00017 | 0.0009 |

   ^1^ From GEE model with robust standard errors, with Bonferroni adjustment. [↑](#endnote-ref-1)
